# Sensitivity Analysis of Mechanical Parameters of Different Rock Layers to the Stability of Coal Roadway in Soft Rock Strata

**DOI:** 10.1155/2013/869040

**Published:** 2013-12-26

**Authors:** Zeng-hui Zhao, Wei-ming Wang, Xin Gao, Ji-xing Yan

**Affiliations:** ^1^State Key Laboratory of Mining Disaster Prevention and Control Cofounded by Shandong Province and the Ministry of Science and Technology, Qingdao, Shandong 266590, China; ^2^College of Civil Engineering of Shandong University of Science and Technology, Qingdao, Shandong 266590, China

## Abstract

According to the geological characteristics of Xinjiang Ili mine in western area of China, a physical model of interstratified strata composed of soft rock and hard coal seam was established. Selecting the tunnel position, deformation modulus, and strength parameters of each layer as influencing factors, the sensitivity coefficient of roadway deformation to each parameter was firstly analyzed based on a Mohr-Columb strain softening model and nonlinear elastic-plastic finite element analysis. Then the effect laws of influencing factors which showed high sensitivity were further discussed. Finally, a regression model for the relationship between roadway displacements and multifactors was obtained by equivalent linear regression under multiple factors. The results show that the roadway deformation is highly sensitive to the depth of coal seam under the floor which should be considered in the layout of coal roadway; deformation modulus and strength of coal seam and floor have a great influence on the global stability of tunnel; on the contrary, roadway deformation is not sensitive to the mechanical parameters of soft roof; roadway deformation under random combinations of multi-factors can be deduced by the regression model. These conclusions provide theoretical significance to the arrangement and stability maintenance of coal roadway.

## 1. Introduction

In recent years, coal industry is tending to transfer to the western area and most of the mining areas are mainly concentrated in Inner Mongolia, Shanxi, Gansu, Ningxia, Xinjiang and so on. The main rock layers of these areas are Jurassic and cretaceous soft rock strata with great thickness due to the special diagenetic environment and depositional history in western China [1–3]. It is difficult for the excavation and maintenance of tunnel in such soft rock strata which shows the poor characteristics of low strength (uniaxial compressive strength *R*_*c*_ < 15 MPa) and weak cementation (argillaceous cementation). Hence, tunnels are mainly arranged in relatively stable coal seam. However, the shape of roadway is often seriously damaged after excavation due to the large deformation of surrounding rock which brings terrible impact on the production safety. Thus, it is urgent to grasp the deformation laws of the tunnel surrounding rock in order to seek reasonable constructing principle and supporting scheme.

Deformation of coal roadway is actually determined by the global mechanical behavior of three body model composed of soft rock and coal seam, which shows obvious structure effect due to the different properties of rock layers. Wang and Feng [4] discussed the influence of side walls strength on floor heave and brought forward the conception of floor heave control through reinforcing the sides in deep tunnel. Based on numerical simulation and site application, Shan et al. [5] found that the overall stability of the roadway could be dramatically improved by increasing the intensity of coal sides. Driad-Lebeau et al., Xu et al., Ortlepp et al., and Procházk [6–9] established some mechanical models of rock-burst in roadway floor considering the occurrence condition and influencing factors, and then analyzed the main factors affecting floor heave which were induced by floor shocking. Furthermore, the large deformation behaviors of compound roof of coal roadway under the action of deep high stress were studied by Sunet al., Xiao et al., Wang et al., Gao et al. and Zhang and Xu [10–14] who held that the deformation force were mainly from the roof pressure and the roof should be treated as a key part to be controlled. All the above results showed the overall structural behavior of tunnel deformation in hard rock strata. However, they are not suitable for soft rock geological environment proposed in this paper.

For a complicated compound structure composed of different rock layers with discrete lithology, it is difficult to establish the relationship between its deformation and influencing factors by an analytical expression. By comparison, the numerical method is more effective. However, large discreteness exists in various mechanical parameters obtained by geological exploration and indoor tests which bring difficulties to the parameters selection for numerical calculation, so the key problem to improve the numerical accuracy is to determine their value scopes and sensitivity according to tunnel deformation. Hou et al., Nour et al., Fenton et al., Gill et al., and Jia et al. [15–19] have carried out some works in this field. However, their conclusions are considered inapplicable to the model contained in this paper because of the difference in formation lithology.

In this paper, a compound system model composed of soft rock and coal seam is established firstly according to the geological conditions and construction characteristics in Xinjiang Ili mine, and then sensitivity analysis of mechanical parameters of each rock layer to the stability of coal roadway is carried out in order to find out the high sensitivity factors which have significant influences on roadway displacement. The conclusions obtained may provide some theoretical principles and optional methods for correct selection of simulation parameters, as well as improving the overall stability of coal roadway.

## 2. System Analysis Model

### 2.1. Physical Model for Coal Roadway in Soft Rock Strata

**Figure 1:**
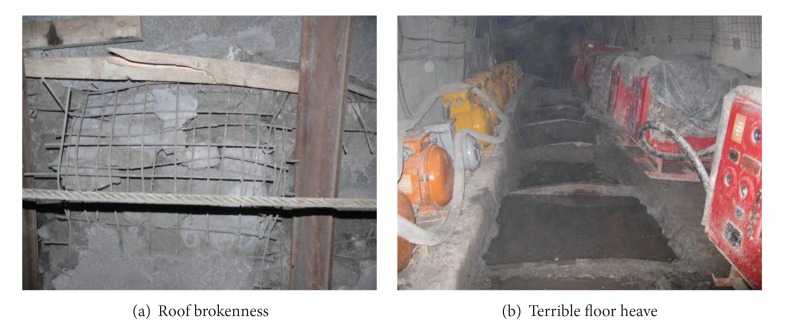
Tunnel failure in engineering site.

The tunnel this paper had chosen is located in Ili forth mine in Xinjiang area. Soft rock strata such as mudstone and sandstone layers with low strength were encountered during the construction of transport tunnel. From the field monitoring data, the rates of roof subsidence and floor heave develop fast after excavation. As shown in Figure 1, terrible mine disaster of roof brokenness and floor heave appear rapidly during construction.

**Figure 2:**
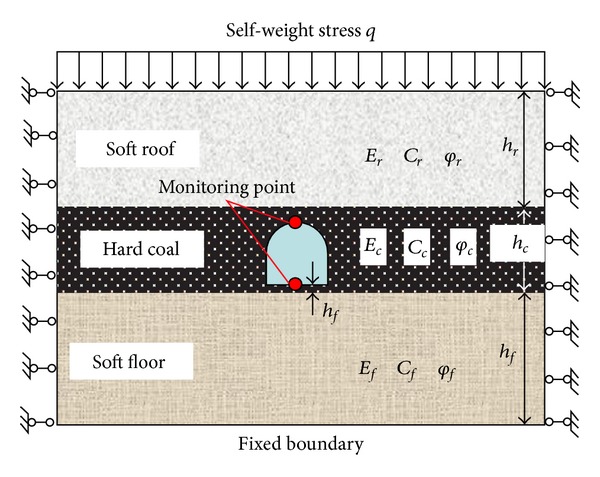
Compound physical model of soft rock and coal seam.

The layout of tunnel is usually passing through coal seam due to the characteristics of low stiffness, being easy to deform, and weak stability of weak cementation soft rock strata. For this, a compound physical model composed of soft rock coal seam was built as shown in Figure 2, where the coal roadway is clamped by upper soft mudstone and lower argillaceous sandstone. *q* is the self-weight stress of overlying strata. The horizontal displacements of left and right boundary lines were restricted along with a fixed bottom. Due to the shallow depth of roadway, only gravity stress and horizontal stress are considered while ignoring the effect of tectonic stress.

### 2.2. Sensitivity Analysis Model

With the features of shallow buried depth and relatively stable coal seam, the damage of side walls is not apparent, and tunnel failures are mainly concentrated on soft roof and floor. As the compound structure shown in Figure 2, the displacements of roof and floor which are labeled as *U*_*r*_ and *U*_*f*_, respectively, were selected as the stability evaluation indexes. Let be the displacement of roof to floor *U* = *U*_*r*_ and *U*_*f*_. For certain strata with specific thickness and buried depth, the main factors influencing the displacement of monitoring points are *α* = {*E*, *C*, *φ*, *h*}, namely, the deformation modulus, cohesion force, and friction angle of each rock layer as well as the depth of coal seam on the bottom of tunnel floor (see in Figure 2). Therefore, the system features can be determined by the following function:

(1) $$\begin{array}{c} U_{r}=f_{1}\left(E_{r},C_{r},\varphi_{r},E_{f},C_{f},\varphi_{f},E_{c},C_{c},\varphi_{c},h\right)\\ U_{f}=f_{2}\left(E_{r},C_{r},\varphi_{r},E_{f},C_{f},\varphi_{f},E_{c},C_{c},\varphi_{c},h\right)\\ U=f\left(E_{r},C_{r},\varphi_{r},E_{f},C_{f},\varphi_{f},E_{c},C_{c},\varphi_{c},h\right), \end{array}$$

where subscripts *r*, *c*, and *f* represent roof, coal seam, and floor, respectively. Distributions of basic parameters and monitoring points are labeled in Figure 2.

**Figure 3:**
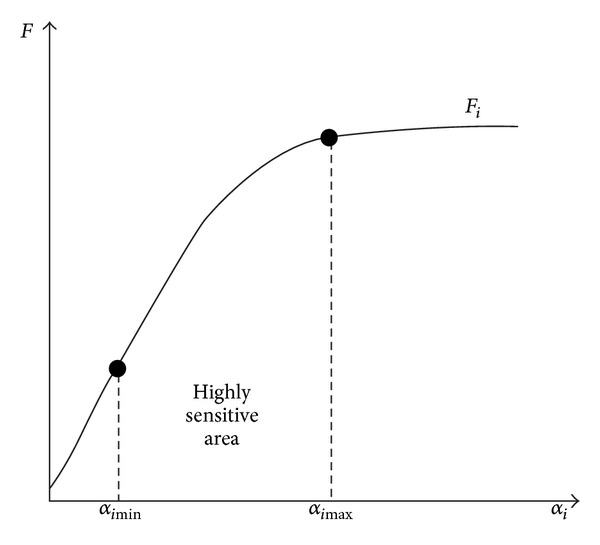
Changes of system characteristic *F* with *α*_*i*_.

Suppose that the system reference state is F* = {*U*_r_*, *U*_f_*, *U*} when the influencing factors are set as base values α* = {E*, C*, φ*, h*}. The deviation trend and degree of system state *F* = {*U*_*r*_, *U*_*f*_, *U*} to reference state F*can be analyzed when the parameter set *α* changes within the scope of its possible levels. Suppose that influencing factors are unitedly represented as *α* = {*α*_1_, *α*_2_, *α*_3_,…, *α*_*n*_}, the sensitivity of system feature *F* to a particular factor *α*_*i*_ can be investigated by making *α*_*i*_ change within its scope, and other factors keep their basic values. Let this system feature be *F*_*i*_ as shown in Figure 3.

Let *α*_*i*_ ∈ {*α*_*i*min⁡_, *α*_*i*max⁡_}. Small changes of parameter *α*_*i*_ may lead to rapid transformation of system feature *F*_*i*_ when *α*_*i*_ is near the value *α*_*i*min⁡_. In other words, *F* is highly sensitive to *α*_*i*_; on the contrary, there is little change of system feature *F* with variation of *α*_*i*_ when *α*_*i*_ is near  *α*_*i*max⁡_ and *F* is insensitive to *α*_*i*_. This implies that it is crucial to make clearly the value ranges of influencing factors and their basic value set in order to accurately analyze the sensitivity of system characteristics *F* to various factors *α*.

Let the system response be *F*_*i*_ ∈ {*F*_*i*min⁡_, *F*_*i*max⁡_} when factor *α*_*i*_ changes within its scope; then, the sensitivity of *F*_*i*_ to *α*_*i*_ can be calculated as follows:

(2) $$\begin{array}{c} S\left(\alpha_{i}\right)=\max\left\{\left(\frac{F_{i\max}-F^{*}}{F^{*}}\right),\left(\frac{F^{*}-F_{i\min}}{F^{*}}\right)\right\}. \end{array}$$

## 3. Numerical Model

### 3.1. Basic Parameters Set

The values of basic parameters set α* = {E*, C*, φ*, h*} are established according to laboratory test results, and all the test specimens were collected from engineering site. Parameters of coal seam thickness *h* at the bottom of tunnel and the changing levels of each factor were designed based on geological observation data. The resulting basic parameters set are shown in Table 1.

### 3.2. Constitutive Model

The test results showed mudstone, argillaceous sandstone, and coal exhibited apparent strain softening properties under low confining pressure, so a strain softening model should be considered according to the intensity attenuation characteristics after postpeak deformation. Thus, compound failure criteria composed of M-C shear failure criteria and tensile failure criteria were employed where M-C yield function was defined as

(3) $$\begin{array}{c} F^{S}=\sigma_{1}-\sigma_{3}\text{ta}{\text{n}}^{2}\left(45^{\circ }+\frac{\varphi }{2}\right)+2c\tan\left(45^{\circ }+\frac{\varphi }{2}\right), \end{array}$$

and tensile failure function was

(4) $$\begin{array}{c} F^{t}=\sigma^{t}-\sigma_{3}, \end{array}$$

where *φ* denotes friction angle, *c* is cohesion force, and *σ* ^*t*^ is the tensile strength.

The strength parameters of each rock layer are changed in linear attenuation with equivalent plastic strain *ε* ^ps^ which can be established by

(5) $$\begin{array}{c} \varepsilon^{\text{ps}}=\frac{1}{\sqrt{2}}\sqrt{{\left(\varepsilon_{1}^{p}-\varepsilon_{m}^{p}\right)}^{2}+{\left(\varepsilon_{m}^{p}\right)}^{2}+{\left(\varepsilon_{3}^{p}-\varepsilon_{m}^{p}\right)}^{2}}\\ \varepsilon_{m}^{p}=\frac{\varepsilon_{1}^{p}+\varepsilon_{3}^{p}}{3}. \end{array}$$

### 3.3. Finite Element Model

**Figure 4:**
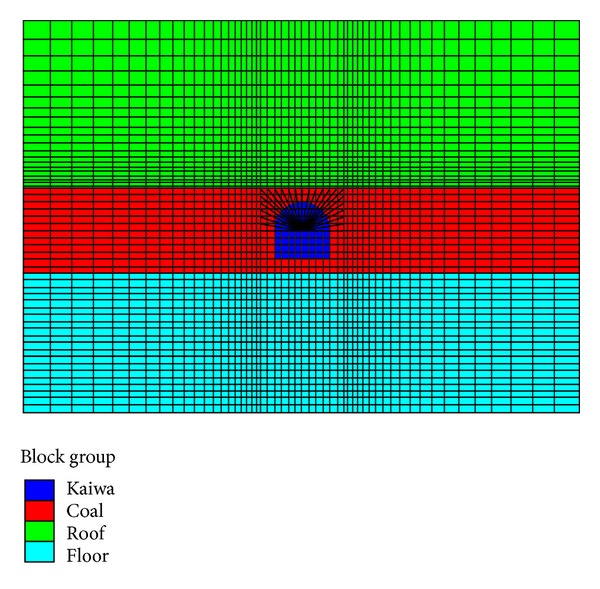
Finite element model of the interbedded structure.

In order to avoid boundary effect, the size of finite element model is set as 40 m × 40 m where the thickness of roof floor and coal seam are *h*_*r*_ = *h*_*f*_ = 15 m and *h*_*c*_ = 10 m, respectively. A finite element model with plane strain assumption was introduced as shown in Figure 4. Some other mechanical parameters were obtained as follows by indoor test: densities of each layer are *ρ*_*r*_ = 2000, *ρ*_*c*_ = 3000, and *ρ*_*f*_ = 1000, respectively, and distributions of Poisson's ratio are *μ*_*r*_ = *μ*_*f*_ = 0.3 and *μ*_*c*_ = 0.2. The span length of coal roadway is 4 m, and the heights of straight wall and arc are both set as 2 m. The value of overlying origin rock stress *q* is 8 MPa according to the result of in situ stress measurement.

## 4. Parameter Sensitivity Analysis

### 4.1. Comparison of Sensitivity Coefficients of Various Factors

**Figure 5:**
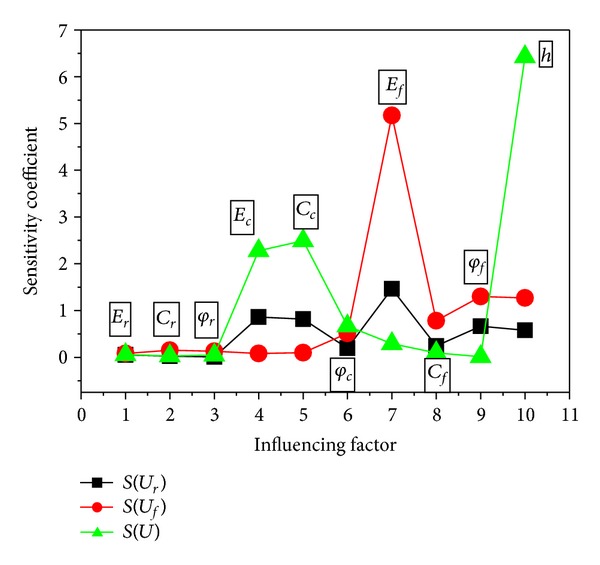
Comparison of various sensitivity coefficients for influencing factors.

Figure 5 and Table 2 showed the calculating results of various sensitivity coefficients for different influencing factors where *S* stands for sensitivity.

Obviously, convergence displacements of roadway are insensitive to the modulus and strength of soft roof, and the level of sensitivity coefficient is *S* < 0.1. On the contrary, the mechanical parameters of soft floor and coal seam have a great effect on tunnel displacements. The rankings of sensitive degree of bed separation displacement *U*_*r*_, floor displacement *U*_*f*_, and total tunnel convergence displacement *U* to each factor were *E*_*f*_ > *E*_*c*_ > *C*_*c*_ > *φ*_*f*_ > *h* > *C*_*f*_ > *φ*_*c*_ > *E*_*r*_ > *C*_*r*_ > *φ*_*r*_, *E*_*f*_ > *φ*_*f*_ > *h* > *C*_*f*_ > *φ*_*c*_ > *C*_*r*_ > *φ*_*r*_ > *C*_*c*_ > *E*_*c*_ > *E*_*r*_, and *h* > *C*_*c*_ > *E*_*c*_ > *E*_*f*_ > *φ*_*c*_ > *C*_*f*_ > *E*_*r*_ > *φ*_*r*_ > *C*_*r*_ > *φ*_*f*_, respectively. Sensitivity coefficients of *U*_*r*_ and *U*_*f*_ to *E*_*f*_ can reach 1.46 and 5.17, respectively, while it reduced to 0.29 for total displacement *U*, and this result further illustrates the overall structure effect of the roadway deformation. Besides, the depth of coal seam *h* at the bottom of roadway also showed great influence on *U*_*f*_ and *U*, the corresponding sensitivity coefficients are 1.27 and 6.43, respectively. The changing laws of system feature *F* with highly sensitive factors were further analyzed in the following section.

### 4.2. System Responses Analysis under the Action of Highly Sensitive Factors

#### 4.2.1. Effect of Roadway Arrangement

**Figure 6:**
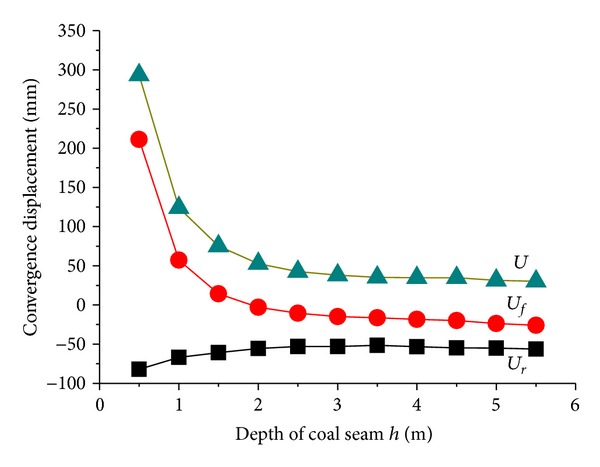
Changes of tunnel displacements with depth of coal seam under floor.

Change law of system feature *F* with depth of coal seam under floor was shown in Figure 6 where *h* was set within the scope of 0.5 m ≤ *h* ≤ 5.5 and other factors were taken their basic values. When *h* ≤ 2 m, *U*_*r*_ and *U*_*f*_ were highly sensitive to *h*. *U*_*r*_ and *U*_*f*_ were sharply increased with decreasing of coal depth under floor, especially in floor heave; opposite tendency appears when *h* ≥ 2 m, *U*_*r*_, and *U*_*f*_ tend to be stable, and the floor begins to sink where no floor heave occurs. The increasing of *h* means the shrinking of coal depth between soft roof and tunnel top surface, and this change has little impact on roof subsidence displacement *U*_*r*_. The above results indicated that it is reasonable to let *h* be 2 m in basic parameters set.

#### 4.2.2. Effect of Mechanical Parameters of Coal Seam

**Figure 7:**
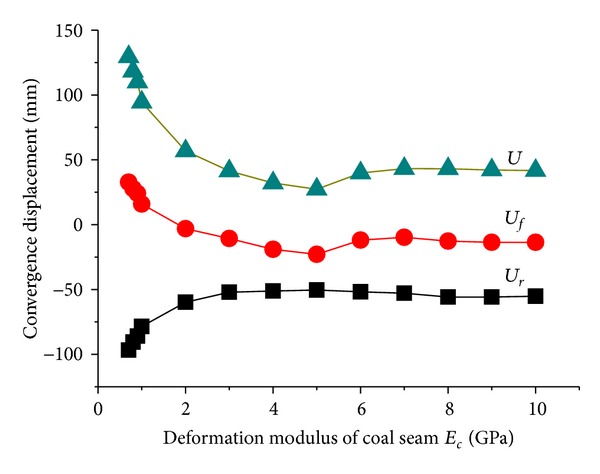
Changes of tunnel displacements with deformation modulus of coal seam.

Figure 7 illustrated the change law of tunnel displacements with deformation modulus *E*_*c*_ of coal seam. While *E*_*c*_ ≤ 2 GPa, *U*_*r*_ and *U*_*f*_ were obviously decreased with the reduce of  *E*_*c*_; if *E*_*c*_ > 2 GPa, *U*_*f*_ was less than 0 and floor heave diminishs, meanwhile roof displacement  *U*_*r*_ tends to be stable. This is because the coal seam becomes “hardening” with the increasing of deformation modulus *E*_*c*_ which result in the large overall compression deformation of soft floor and disappearance of floor heave.

**Figure 8:**
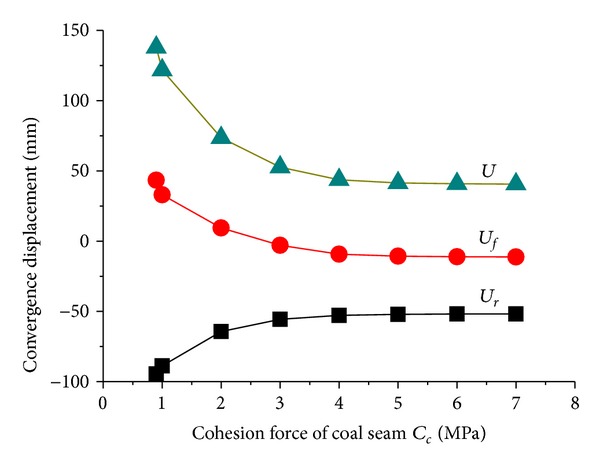
Changes of tunnel displacements with strength parameters of coal seam.

Figure 8 revealed the effect of cohesion force *C*_*c*_ of coal seam on tunnel convergence displacement. When *C*_*c*_ ≤ 3 MPa, side walls of coal seam suffered shear failure, and *U*_*r*_ and *U*_*f*_ are both at high levels. While *C*_*c*_ > 3 MPa, only elastic zone occurs in the side walls due to the increasing of coal strength, and tunnel displacements become stable. That is to say, the deformation of roof and floor is closely related to the stability of side walls of coal seam which further reflect the integrity of the roadway stability. Besides, influence of cohesive force is greater than the friction angle.

#### 4.2.3. Effect of Mechanical Parameters of Soft Floor

**Figure 9:**
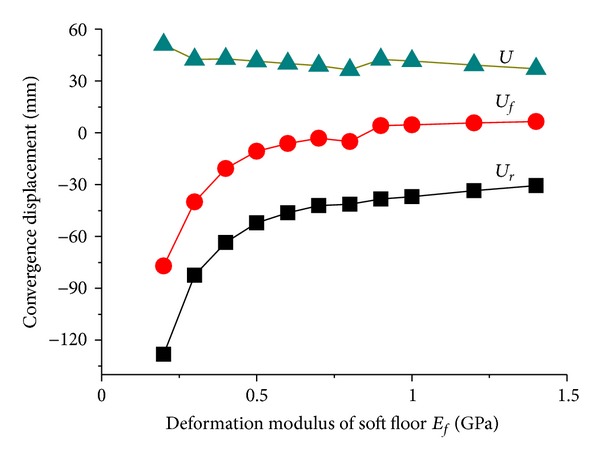
Changes of tunnel displacements with deformation modulus of soft floor.

Figure 9 showed the influence of deformation modulus *E*_*f*_ on tunnel displacement. When *E*_*f*_ ≤ 1 MPa, the soft floor produced larger overall compression deformation due to the weak stiffness which results in the overall declination of surrounding rock. Because of *U*_*f*_ < 0, no floor heave occurs. The relationship of *U*_*r*_ > *U*_*f*_ means that the floor stiffness has a great effect on roof displacement. With the increasing of *E*_*f*_, floor heave occurs and roof sag displacement tends to be stable. The results show that *E*_*f*_ has a little impact on total convergence displacement *U*.

**Figure 10:**
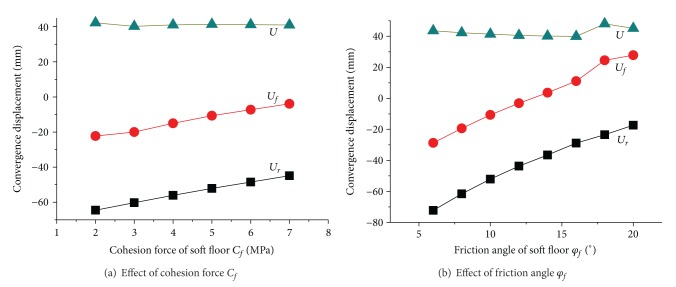
Changes of tunnel displacements with strength parameters of soft floor.

The change law of convergence displacement with strength parameters of *C*_*f*_ and *φ*_*f*_ was described in Figure 10(a) and Figure 10(b), respectively. A large area that suffered plastic shear failure may be produced in soft floor when the strength parameters were at a low level, and subsidence of roof and floor appears. With the increasing of strength levels, failure area decreased in floor which began to transfer to the corner area of roadway, and roof displacement tends to decrease while floor sinking turns to floor heave. Actually, effect of friction angle *φ*_*f*_ is greater than cohesive force *C*_*f*_, but the two both have no significant influence on the total convergence displacement *U*.

### 4.3. A Multifactors Regression Model of the System

The regression model of system response *F* under the action of a single factor *α*_*i*_ can be built by curve regression as follows:

(6) $$\begin{array}{c} F=a_{0}+b\cdot \exp\left(c\cdot \alpha_{i}\right), \end{array}$$

where *a*_0_, *b*, and *c *are the regression coefficient, respectively.

Single factor analysis can only determine the influence law of a separate factor on the stability of surrounding rock, so it is difficult to establish the cross-influences of different parameters on the system feature. Thus, it is necessary to establish a multi-factors regression model for the system in order to make clearly the system sensitivity while multiple parameters change at the same time. For this, the system regression model is set as follows based on the influencing laws of each factor obtained in Section 4.2:

(7) Ur=a0+a1h−+a2E−c+a3C−c+a4φ−c+a5E−f +a6C−f+a7φ−f+a8E−r+a9C−r+a10φ−rUf=b0+b1h^+b2E^c+b3C^c+b4φ^c +b5E^f+b6C^f+b7φ^f+b8E^r+b9C^r+b10φ^r

in which *a*_*i*_, *b*_*i*_  (*i* = 0,1, 2,…, 10) stand for undetermined coefficients. The equivalent mechanical parameters have the following forms:

(8) $$\begin{array}{c} \overset{-}{h}=e^{-1.59h},{\;\;\overset{-}{E}}_{c}=e^{-1.64E_{c}},\\ {\overset{-}{C}}_{c}=e^{-1.12C_{c}},\;\;{\overset{-}{\varphi }}_{c}=e^{-0.08\varphi_{c}},\\ {\overset{-}{E}}_{f}=e^{-5.82E_{f}},{\;\;\overset{-}{C}}_{f}=e^{-0.034C_{f}},\\ {\overset{-}{\varphi }}_{f}=e^{-0.053\varphi_{f}},{\;\;\overset{-}{E}}_{r}=e^{-2.48E_{r}},\\ {\overset{-}{C}}_{r}=e^{0.93C_{r}},\;\;{\overset{-}{\varphi }}_{r}=e^{-0.06\varphi_{r}},\;\;\hat{h}=e^{-2.06h},\\ {\hat{E}}_{c}=e^{-1.23E_{c}},\;\;{\hat{C}}_{c}=e^{-0.87C_{c}},\\ {\hat{\varphi }}_{c}=e^{-0.0018\varphi_{c}},\;\;{\hat{E}}_{f}=e^{-5.56E_{f}},\;\;{\hat{C}}_{f}=e^{0.86C_{f}},\\ {\hat{\varphi }}_{f}=e^{-0.0036\varphi_{f}},\;\;{\hat{E}}_{r}=e^{-0.53E_{r}},\\ {\hat{C}}_{r}=e^{4.43C_{r}},{\;\;\hat{\varphi }}_{r}=e^{0.08\varphi_{r}}. \end{array}$$

By substitution, undetermined coefficients can be established by using linear multiple regression analysis. Finally, regression result of the system response model is

(9) Ur=1227.1−69.75e−1.59h−141.3e−1.64Ec−113.5e−1.12Cc   −76.2e−0.08φc−294.9e−5.82Ef−1193.3e−0.034Cf   −144.9e−0.053φf−3.63e−2.48Er+1.15e0.93Cr   −1.19e−0.06φrUf=634.2+574.7e−2.06h+99.7e−1.23Ec+106.8e−0.87Cc   +358.4e−0.0018φc−251.3e−5.56Ef+30.7e0.86Cf     −1149.3e−0.0036φf−4.46e−0.53Er   +6.27e4.43Cr+0.018e0.08φr.

The unit type of each mechanical parameter is in line with Table 1. The system sensitivity can be investigated by 8 under the action of multi-factors.

**Figure 11:**
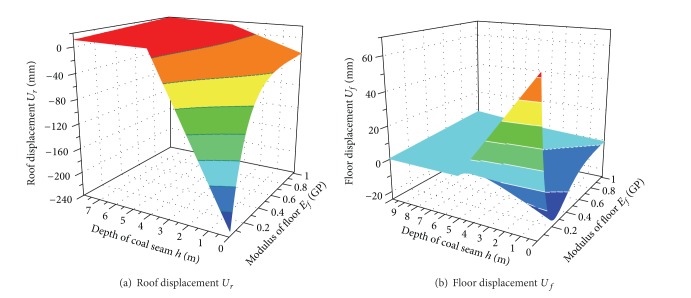
System responses with changes of multiple-factors.

Figure 11 showed the contour lines of system response when the elastic modulus *E*_*f*_ and *h* were changed simultaneously within their scope. It is easy to obtain the convergence displacements under the arbitrary combination of *h* and *E*_*f*_. Similarly, the displacement responses can also be quickly established when other parameters change. The regression model provides theoretical basis for determining the optimal parameter combination which can minimize the tunnel displacement.

## 5. Conclusions

1. The tunnel displacement is highly sensitive to the depth of coal seam under soft floor. On the condition of specified thickness of each layer, the deformation of roof and floor tend to be stable when *h* ≥ 2 m, so it should be fully considered in the arrangement of coal roadway.
2. The mechanical parameters of coal seam and soft floor have significant influence on tunnel displacement; the direction of floor displacement will be changed by enhancing the stiffness of coal seam which can effectively prevent and control floor heave as well as decrease the roof subsidence; the damage state of side walls is determined by strength parameters of coal seam; therefore, the overall stability of roadway can be improved by the reinforcement of side walls. The roof subsidence can be obviously reduced by increasing the elastic modulus of floor. The floor displacement can be dramatically decreased by improving the floor strength which is closely related to the shear failure area of soft floor. The tunnel displacement is not sensitive to the mechanical parameters of soft floor.
3. The multifactors regression model laid a theoretical foundation for seeking the best parameter combination and providing reasonable supporting to control the deformation of this kind of roadway.

## Figures and Tables

**Table 1:** Basic value of parameters and their changing levels.

Factor	Basic value	Scope	Distribution	Level
*E*_*r*_/GPa	0.7	0.5–1	Uniform	6
*C*_*r*_/MPa	6	4–9	Uniform	6
φ_r_/°	15	10–20	Uniform	5
*E*_*f*_/GPa	0.5	0.3–0.7	Uniform	5
*C*_*f*_/MPa	5	2–7	Uniform	6
φ_*f*_/°	10	5–15	Uniform	10
*E*_*c*_/GPa	3	1–6	Uniform	6
*C*_*c*_/MPa	4	2–7	Uniform	6
φ_c_/°	35	30–40	Uniform	5
*h*/m	2	0.5–5.5	Uniform	10

**Table 2:** Calculating results of sensitivity coefficient.

Factor	Convergence displacement		
	*U*_*r*_	*U*_*f*_	*U*
*E*_*r*_/GPa	0.04865	0.0792	0.05695
*C*_*r*_/MPa	0.02308	0.152	0.02886
φ_r_/°	0.00808	0.1288	0.0443
*E*_*c*_/GPa	0.86019	0.0832	2.27646
*C*_*c*_/MPa	0.81654	0.1	2.49114
φ_c_/°	0.195385	0.5128	0.66785
*E*_*f*_/GPa	1.46442	5.1704	0.29165
*C*_*f*_/MPa	0.24058	0.7808	0.09392
φ_f_/°	0.66615	1.2984	0.01114
*h*/m	0.576731	1.2712	6.42532
